# A Rare Non Urologic Cause for Urinary Retention; Report of 2 Cases

**DOI:** 10.5812/numonthly.6406

**Published:** 2013-03-30

**Authors:** Seyed Mohammadreza Rabani

**Affiliations:** 1 Beheshti Teaching Hospital, Yasuj University of Medical Sciences, Yasuj, IR Iran

**Keywords:** Hymen, Imperforate, Hematocolpos, Adolescent, Abdominal Pain, Hydronephrosis

## Abstract

Although Imperforate hymen is a rare condition, it is the most common obstructive anomaly of the female genital tract. The early diagnosis of this condition requires a high index of suspicion in newborns and in females without a history of menarche. Hydronephrosis is a known but rare complication of an imperforate hymen. Hereby we preset 2 cases of premenstrual adolescent with urinary retention and bilateral moderate to severe hydroureteronephrosis.

## 1. Background

Imperforate hymen occurs in approximately 1 in 10000 to 1 in 1000 females, although true incidence is difficult to obtain, familial cases have been reported in the literature but are more often sporadic (1-3). In spite of the rarity of this entity, imperforate hymen is the most common obstructive anomaly of the female genital tract, usually results from the vaginal plate that is not canalized during fetal development (4). The clinical presentations of these patients may range from an incidental finding on physical examination of an asymptomatic patient, to findings discovered froman evaluation for primary amenorrhea or cyclic abdominal pain and even rarely for urinary retention. In differential diagnosis of an adolescent girl with abdominal pain and voiding dysfunction and negative history of previous menstruation the diagnosis of imperforate hymen should be considered. Hereby we present 2 cases of imperforated hymen presenting with urologic symptoms.

## 2. Case Presentations

### 2.1. Case 1

**Figure 1.**
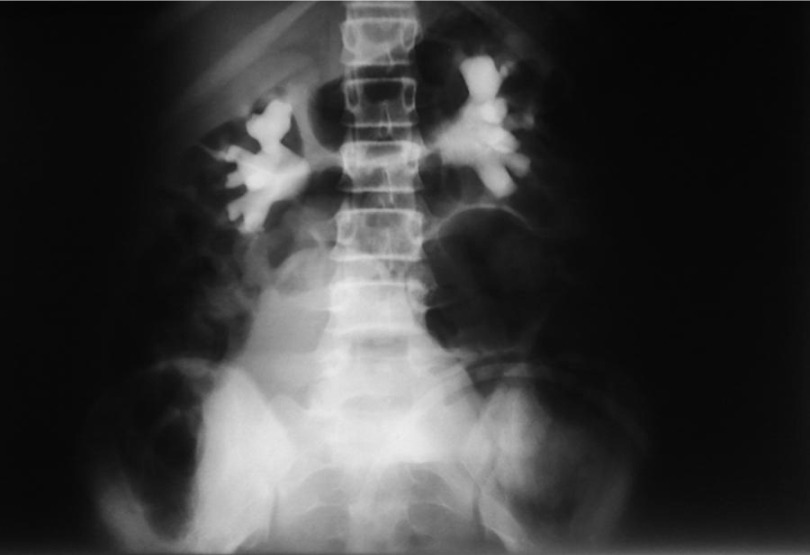
IVP, 10 Minutes Film

**Figure 2.**
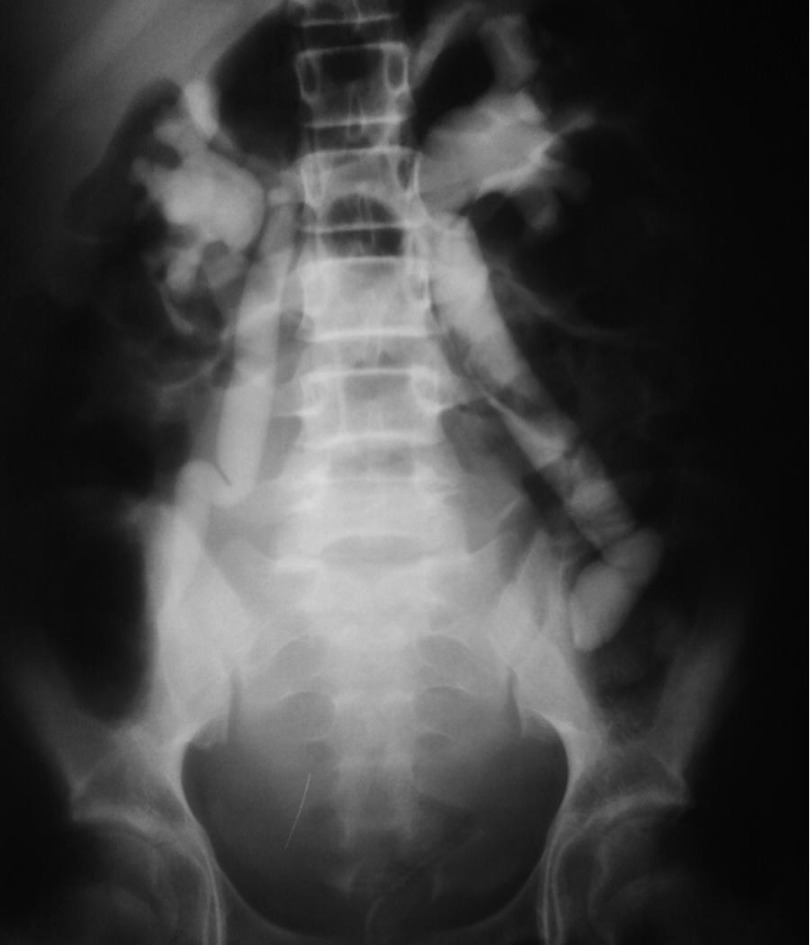
IVP, 20 Minutes Film

**Figure 3.**
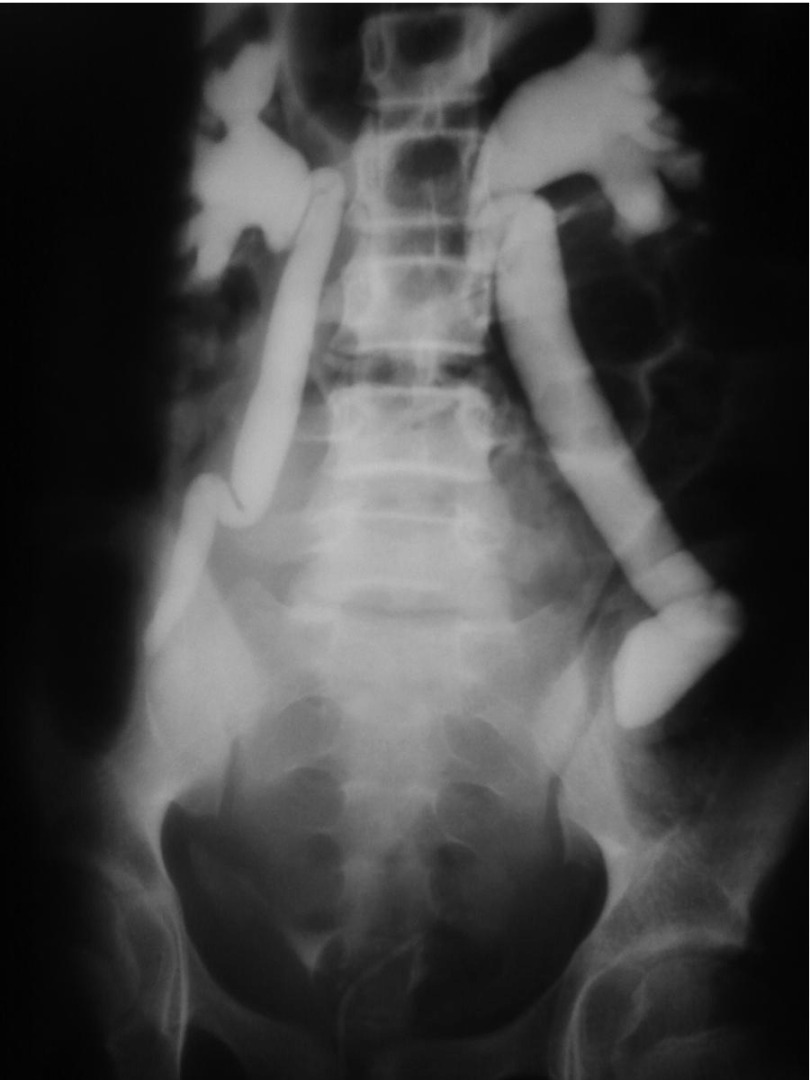
30 Minutes Film

An 11-year- robust girl presented with sudden onset abdominal pain and inability to void. She was admitted in emergency room. She had also a history of vague cyclic abdominal pain from about 6 months before her admission. On physical examination, the abdomen was tendered and distended, more severe on lower abdomen. According to history of inability to void and distended abdomen, an indwelling catheter was inserted and about 200 mL clear urine drained, but the abdomen was still distended. Abdominal and pelvis sonography was done that unfortunately in the presence of indwelling catheter, reported a very distended, elongated and irregular bladder, accompanied with bilateral moderate to severe hydronephrosis and dilated, tortuous ureters. Requested Lab. Data except for renal function tests (BUN = 26, Cr = 1.2), all were in almost normal ranges. Intavenous urography (IVP) was requested that showed bilateral hydroureteronephrosis with mass effects caused by soft tissue density (Figures 1, 2 and 3). Again a physical examination based on IVP findings was done under general anesthesia, that obviously confirmed the diagnosis of imperforate hymen. Considering the cultural and religious believes a hymenotomy was designed that its appearance was finally like an intact hymen. This was achieved using a subtle, low energy cauterization of the hymen after suction drainage of more than 1000 mL of red, tarry blood. The patient was followed after one and 3 months. In both follow ups ultrasonography of abdomen and pelvis were normal without any evidence of parenchymal damage or residual hydronephrosis. Renal function tests were normal (BUN = 11, Cr = 0.6). Menstruation in 3 months happened only once and was without difficulty and the hymen general appearance was like a normal, unmanipulated hymen.

### 2.2. Case 2

A 13-year-old rural girl was admitted to the emergency room due to lower abdominal pain and urinary retention. She had a history of repeated abdominal pain from about 6 months before admission but without menarche. On physical examination the thin abdomen was significantly distended and mildly tendered, more severe on the lower abdomen and the secondary sexual characteristics were present. All Routine laboratory examinations in the emergency room were in normal ranges. Abdominal and pelvis ultrasonography reported urinary retention, mild bilateral hydroureteronephrosis, mild dilatation of the uterus, significant dilatation and elongation of the vagina and suggested hematocolpos. The patient refused physical examination of the vagina even by a female nurse. Using the experiences of the first case, she was taken to the operating room and under general anesthesia, the examination revealed the typical shape of imperforate hymen. A Foley catheter was inserted and the hymenotomy was done similar to case 1. 3 months follow up visits revealed normal renal function tests, normal sonography and uneventful mensrtuation. She refused physical examination of the treated hymen in follow up visits.

## 3. Discussion

Imperforate hymen is a rare congenital anomaly of female genital tract in which the vagina has not an opening in its distal part for draining the usual mucosal secretions of the genital tract and menstrual bleeding. This condition causes collection behind the imperforat hymen and the menarche may be the first trigger for developing symptoms. So it is easy to imagine that we may encounter an adolescent girl with a history of abdominal pain without menarche and in physical examination of the introitus in most cases the diagnosis will be made without using any laboratory or paraclinical technique. On the other hand these patients are easily missed without taking a thorough history and a proper physical examination. The syndrome known as imperforate hymen with hematocolpos has been recognized for centuries and yet is seldom diagnosed by the physician who first sees the patient (5). In our first case we encountered a complicated case with unusual presentation regarding age (only 11-year-old), tendered and distended abdomen with urinary retention and the misleading sonography. In this case the IVP finding of large soft tissue density in the presence of a Foley catheter was the leading cause for diagnosis. It is also interesting that the nurse who inserted the Foley catheter could not understand the abnormal situation of the introitus, while the typical bluish and bulged imperforate hymen was enough for diagnosis. In a review of 65 cases, patients had an average age of 14 years at presentation and Hematocolpos was a constant finding (6). Treatment of this condition is hymenotomy with different methods. A simple linear, cruciate, or plus hymenotomy is preferred by some and others prefer resection of the membrane. In both methods using a catheter in postoperative period for a few days to weeks were suggested. Suturing the labia of treated hymen was also suggested to prevent recurrence of the situation. We used a subtle, low energy cautery device after suction drainage by a small suction probe. The aim of our procedure was to create a normal appearing hymen membrane with enough opening to prevent recurrence of the condition and this was based on cultural beliefs of our people and in short term follow up we achieved this aim.

## 4. Conclusion

Imperforate hymen is a rare condition, but should be considered when dealing with premenarcheal adolescent females with voiding dysfunction and lower abdominal symptoms.
